# Supplementary material for: Drug repurposing for aging research using model organisms
Source: Aging Cell. 2017 Jun 16;16(5):1006–15. doi: 10.1111/acel.12626 (PMC5595691; doi:10.1111/acel.12626)
Supplement: Supplementary file 7 — Data S1 Zip‐Archive of all report cards. [file ACEL-16-1006-s007.zip › RC_PPF.pdf]

# PPF

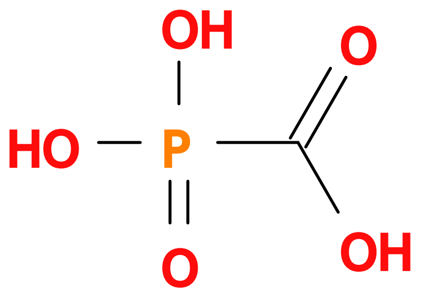


**Database identifiers**

| | |
|---|---|
| ChEMBLCompound | CHEMBL666 |
| DrugBank | DB00529 |
| CHEBI | 127780 |

## Ranking

| | Rank | Score |
|---|---|---|
| Drosophila | 495/697 | 0.281 |
| C. elegans | 251/591 | 0.222 |

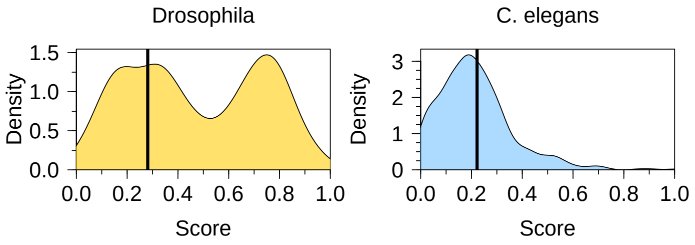


| | Ageing implication | Domain conservation | Binding site conservation | Binding affinity | Bioavailability | Lipinski | Promiscuity | Purchasability | Drug approval | **Total** |
|---|---|---|---|---|---|---|---|---|---|---|
| Drosophila | 0.36 | 0.942 | 1.0 | 0.267 | (0.9) | 0.0 | -0.0 | 0.1 | 0.1 | 0.281 |
| C. elegans | 0.36 | 0.936 | 1.0 | 0.267 | 0.25 | 0.0 | -0.0 | 0.1 | 0.1 | 0.222 |

## Names

- **phosphonoformic acid**
- carboxyphosphonic acid
- Dihydroxyphosphinecarboxylic Acid Oxide
- foscarnet
- Foscavir
- phosphonomethanoic acid

## Roles

antiviral drug, HIV-1 reverse transcriptase inhibitor, sodium-dependent Pi-transporter inhibitor

## Status

| | |
|---|---|
| Approved drug (according to ChEMBL) | Yes |
| Administration Route | Intravenous drip |
| Number of Rule of 5 violations | 0 |
| Binding affinity to original target in log units (RF-Score prediction) | 3.99 |
| Burns *C. elegans* bioavailability prediction | -2.08 |

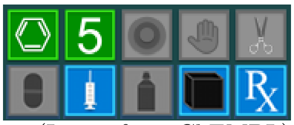

(Image from ChEMBL)

## Medical Information

**Indicatation:** For the treatment of CMV retinitis in patients with acquired immunodeficiency syndrome (AIDS) and for treatment of acyclovir-resistant mucocutaneous HSV infections in immunocompromised patients.
**Mechanism of action:** Foscarnet exerts its antiviral activity by a selective inhibition at the pyrophosphate binding site on virus-specific DNA polymerases at concentrations that do not affect cellular DNA polymerases.
**Toxicity:** Oral, rat LD50: >2,000 mg/kg. Signs of overdose include renal impairment.
**Metabolism:** Not metabolized.
**Retail:** AstraZeneca Inc., Hospira Inc., Spectrum Pharmaceuticals
Sodium phosphonoformate powder - 286.3 USD / g
Foscarnet 24 mg/ml infus bttl - 0.37 USD / ml
Foscavir 24 mg/ml infus bottle - 0.43 USD / ml

(Information from DrugBank)

## Compound Target Characteristics

### Phosphoenolpyruvate carboxykinase, cytosolic [GTP]

Best gene implication in ageing for this target family came from gene Q9Z2V4 via mapping the annotation from Ensembl ENSMUSG00000027513 via mapping the annotation from EntrezGene 18534 via mapping the annotation from GenAgeModels 0497 annotated in GenAge release 17.

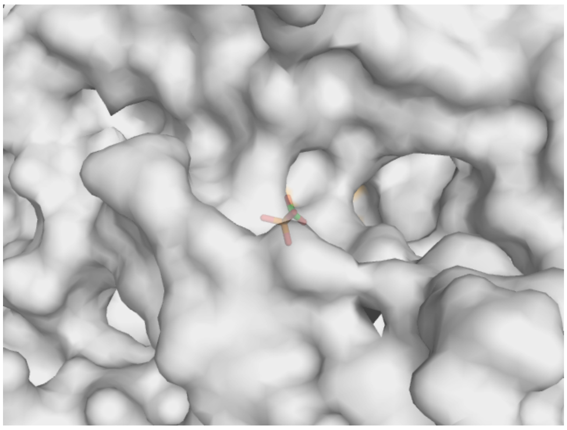

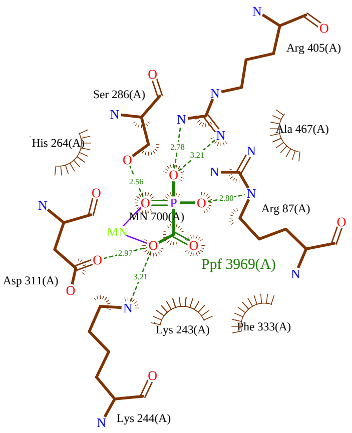

| protein | amino acids contacts (binding site) |
| --- | --- |
| PDB:2rk8:chainA:P07379 | R K K H S D F R A |
| sp:P35558:PCKGC_HUMAN | R K K H S D F R A |
| sp:P07379:PCKGC_RAT | R K K H S D F R A |
| sp:Q9Z2V4:PCKGC_MOUSE | R K K H S D F R A |
| tr:A8DYI3:A8DYI3_DROME | R K K H S D F R A |
| tr:Q7JXB5:Q7JXB5_DROME | R K K H S D F R A |
| sp:P20007:PCKG_DROME | R K K H S D F R A |
| tr:Q7JKI1:Q7JKI1_CAEEL | R K K H S D F R A |
| tr:Q7JKI3:Q7JKI3_CAEEL | R K K H S D F R A |
| tr:Q8ITY0:Q8ITY0_CAEEL | R K K H S D F R A |
| tr:Q7JKI2:Q7JKI2_CAEEL | R K K H S D F R A |
| tr:O44906:O44906_CAEEL | R K K H S D F R A |
| tr:O02286:O02286_CAEEL | R K K H S D F R A |

| | whole protein | | domain-based | | contact-based | |
| --- | --- | --- | --- | --- | --- | --- |
| protein | ident | simil | ident | simil | ident | simil |
| PDB:2rk8:chainA:P07379 | 1.0 | 1.0 | 1.0 | 1.0 | 1.0 | 1.0 |
| sp:P35558:PCKGC_HUMAN | 0.91 | 0.98 | 0.91 | 0.98 | 1.0 | 1.0 |
| sp:P07379:PCKGC_RAT | 1.0 | 1.0 | 1.0 | 1.0 | 1.0 | 1.0 |
| sp:Q9Z2V4:PCKGC_MOUSE | 0.98 | 0.99 | 0.97 | 0.99 | 1.0 | 1.0 |
| tr:A8DYI3:A8DYI3_DROME | 0.6 | 0.8 | 0.62 | 0.83 | 1.0 | 1.0 |
| tr:Q7JXB5:Q7JXB5_DROME | 0.62 | 0.85 | 0.65 | 0.88 | 1.0 | 1.0 |
| sp:P20007:PCKG_DROME | 0.61 | 0.84 | 0.65 | 0.88 | 1.0 | 1.0 |
| tr:Q7JKI1:Q7JKI1_CAEEL | 0.47 | 0.72 | 0.51 | 0.76 | 1.0 | 1.0 |
| tr:Q7JKI3:Q7JKI3_CAEEL | 0.55 | 0.82 | 0.58 | 0.85 | 1.0 | 1.0 |
| tr:Q8ITY0:Q8ITY0_CAEEL | 0.5 | 0.73 | 0.54 | 0.78 | 1.0 | 1.0 |
| tr:Q7JKI2:Q7JKI2_CAEEL | 0.56 | 0.84 | 0.59 | 0.87 | 1.0 | 1.0 |
| tr:O44906:O44906_CAEEL | 0.54 | 0.81 | 0.58 | 0.86 | 1.0 | 1.0 |
| tr:O02286:O02286_CAEEL | 0.54 | 0.82 | 0.59 | 0.87 | 1.0 | 1.0 |

**Pepck (UniProt:P20007) annotation**

**Function**: Catalyzes the conversion of oxaloacetate (OAA) to phosphoenolpyruvate (PEP), the rate-limiting step in the metabolic pathway that produces glucose from lactate and other precursors derived from the citric acid cycle.
**Cofactor**: Mn(2+)Note=Binds 1 Mn(2+) ion per subunit. ;
**Pathway**: Carbohydrate biosynthesis; gluconeogenesis.
**Subunit**: Monomer.

(Information from UniProt)
